# Reversible temporal artery halo sign in polymerase chain reaction–confirmed herpes zoster ophthalmicus without corticosteroid exposure

**DOI:** 10.1093/rap/rkag104

**Published:** 2026-08-17

**Authors:** Howard Jie Yang, Emily Yiming Lin, Rajeev Deva, Eric Morand

**Affiliations:** Department of Rheumatology, Monash Health, Clayton, VIC, Australia; Department of Rheumatology, Monash Health, Clayton, VIC, Australia; Faculty of Medicine, Nursing and Health Sciences, Monash University, Melbourne, VIC, Australia; Department of Radiology, Monash Health, Clayton, VIC, Australia; Department of Rheumatology, Monash Health, Clayton, VIC, Australia; Faculty of Medicine, Nursing and Health Sciences, Monash University, Melbourne, VIC, Australia

Key message

- Herpes zoster ophthalmicus can produce a temporal artery ultrasound halo sign and mimic giant cell arteritis, highlighting the importance of clinical and microbiological correlation.

Dear Editor, Giant cell arteritis (GCA) is an autoimmune vasculitis affecting medium- and large-sized arteries [1]. Temporal artery ultrasound (TAUS) is increasingly used as a first-line investigation for GCA. The hypoechoic circumferential ‘halo sign’ has been reported to demonstrate high specificity and reasonable sensitivity [2]. Herpes zoster ophthalmicus (HZO) occurs when the varicella-zoster virus is reactivated and affects the ophthalmic division of the fifth cranial nerve, and is an important differential diagnosis for GCA in older adults presenting with an acute unilateral headache [3].

We describe a case of HZO associated with bilateral halo signs on TAUS, with dynamic radiographic resolution following antiviral therapy alone, without corticosteroid administration.

A 69-year-old woman presented to the emergency department on two occasions. On her first presentation, she had a left-sided headache, non-claudicant jaw pain and a left temporal erythematous, papular eruption without vesicles. Her past history included hypertension, type 2 diabetes and a distant history of endometrial cancer in remission. GCA with concomitant simple skin infection was considered, prompting TAUS.

The TAUS demonstrated bilateral increased intimal thickness with hypoechoic circumferential non-compressible halos consistent with arteritis. On the right, halo thickness measured 0.5 mm; on the left, halo thickness measured 0.7 mm (Fig. 1). Colour Doppler imaging demonstrated reduced luminal calibre. There was no evidence of axillary or common carotid artery involvement.

**Figure 1:**
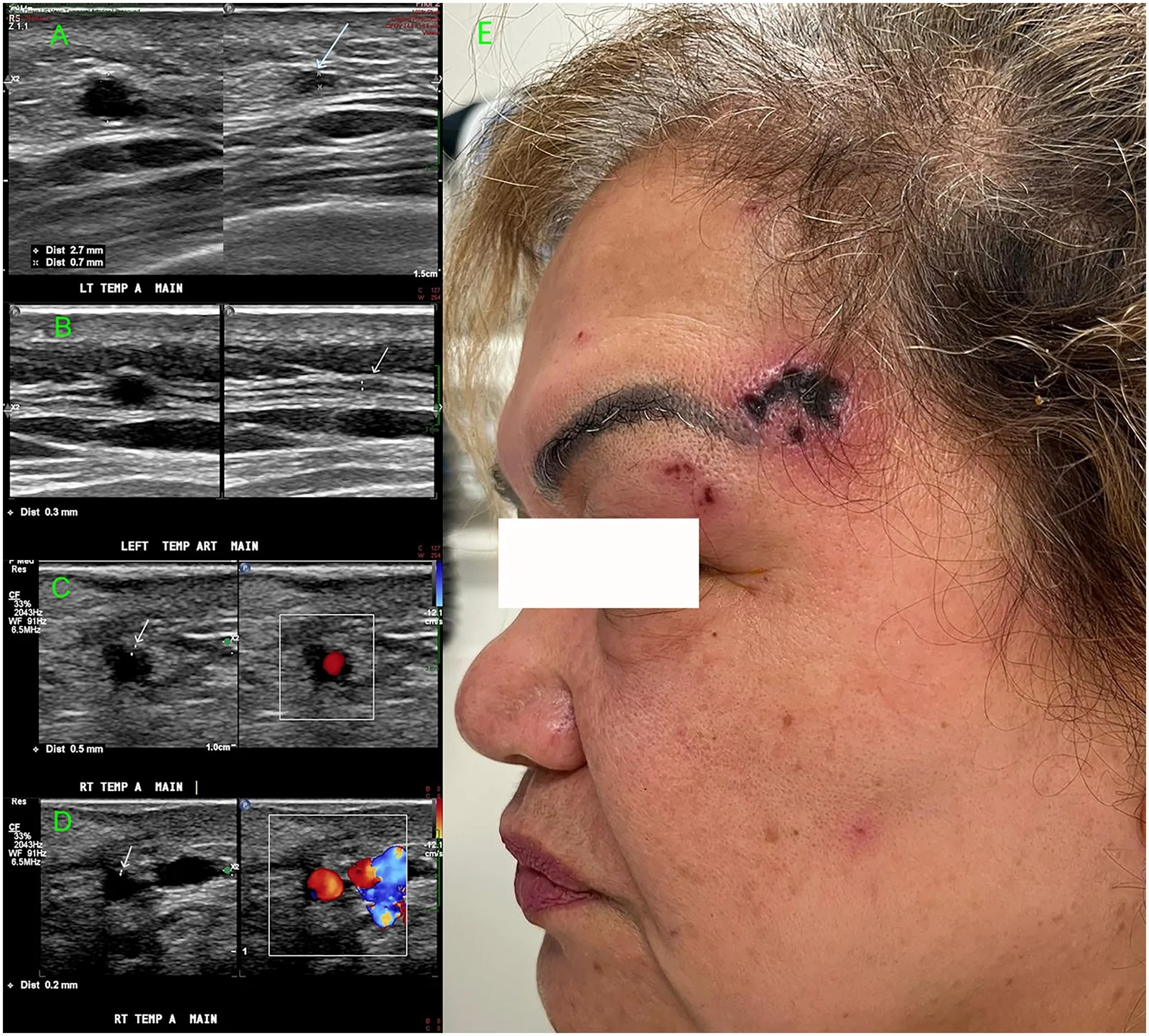
Ultrasound findings and clinical photograph. Transverse ultrasound demonstrating hypoechoic wall thickening with a ‘halo sign’ (white arrow) measuring 0.7 mm in the left (A) and 0.5 mm in the right (C) superficial temporal arteries. Follow-up ultrasound demonstrating resolution of intimal thickening and normal luminal calibre after 9 months in the left (B) and after 2 months in the right (D) temporal arteries. All images were acquired on a Philips Epiq 7 ultrasound machine with a high-resolution linear-array transducer, 7.0–15.0 MHz. Clinical photograph demonstrating a unilateral vesicular eruption with crusted erosions over the left ophthalmic trigeminal distribution consistent with herpes zoster ophthalmicus (E)

The patient was recalled the next day, and the rheumatology unit was consulted. On targeted examination, she had developed clustered erosions involving the left supraorbital and lateral eyebrow region, with a distribution consistent with the ophthalmic division of the trigeminal nerve (Fig. 1). Temporal arteries were palpable, non-tender and without bruits.

There was no scalp tenderness, visual disturbance, constitutional symptoms, limb claudication or polymyalgia rheumatica–type symptoms. Ophthalmological assessment, including fluorescein staining was unremarkable.

Laboratory investigations included an erythrocyte sedimentation rate (ESR) of 13 mm/h and C-reactive protein (CRP) of 2.8 mg/l. Polymerase chain reaction (PCR) testing of skin lesions confirmed varicella zoster virus infection, establishing the diagnosis of herpes zoster ophthalmicus.

Given the absence of clinical and biochemical features supporting GCA and the presence of PCR-confirmed HZO, corticosteroid therapy was not initiated. Given evolving periocular lesions and atypical vascular imaging findings, the patient was treated with oral valaciclovir 1000 mg three times daily for 2 weeks. Her headache completely resolved following completion of antiviral therapy.

Repeat TAUS after 2 months demonstrated resolution of the halo sign on the right side, with only minimal residual thickening (Fig. 1). The left temporal artery showed improvement but persistent mild thickening.

A third TAUS performed in October 2025 demonstrated complete bilateral resolution (Fig. 1). All examined arteries were compressible, with no residual wall thickening, thrombus or occlusion.

TAUS has become increasingly integrated into the diagnostic evaluation of GCA since its inception [2]. The characteristic hypoechoic circumferential ‘halo sign’ reflects arterial wall oedema and intima-media thickening [2]. In GCA, these arise from granulomatous inflammation involving the media and intima, disruption of the internal elastic lamina and subsequent intimal hyperplasia leading to luminal narrowing [1].

VZV has a recognised tropism for arterial tissue [4]. The virus is known to spread transaxonally to adjacent arterial walls, causing vasculopathy [4]. Furthermore, VZV has been identified in temporal artery specimens in patients initially diagnosed with GCA [5]. A causal link between these two pathologies remains controversial due to the ubiquitous nature of VZV and conflicting data [1, 5]. Given that the patient in our case experienced a complete clinical response without targeted GCA therapy, the sonographic findings are favoured to reflect either VZV vasculopathy or periarterial inflammation. The contralateral findings may suggest a degree of immune-mediated inflammation beyond the clinically apparent dermatome. This also suggests that VZV infection can mimic, rather than cause, GCA.

Despite the association between VZV infection and GCA, reports describing an ultrasound halo sign in the context of VZV infection are rare. A review of the literature identified two documented cases of a temporal artery halo sign associated with VZV infection [6, 7].

Teodoro *et al.* [6] described a patient with a positive TAUS halo sign in the context of VZV infection, with subsequent biopsy demonstrating VZV antigen within the temporal artery (Supplementary Table S1). Despite also recording resolution of the halo sign, this outcome was confounded by concurrent corticosteroid exposure [6]. Furthermore, an incomplete clinical response to antiviral therapy alone suggested possible concurrent GCA with superimposed VZV infection [6].

Tsai *et al.* [7] reported a patient with HZO and a positive TAUS who was also pre-emptively treated with corticosteroids (Supplementary Table S1). However, halo resolution was not demonstrated as the patient progressed to temporal artery biopsy, and viral staining of biopsy tissue was negative, leaving uncertainty as to the precise aetiology of radiographic findings [7].

Although a bilateral halo sign is often regarded as highly specific, this case illustrates that virus-associated arterial inflammation may produce indistinguishable sonographic findings. Clinical, laboratory and imaging data should therefore be integrated when evaluating suspected GCA.

## Supplementary Material

rkag104_Supplementary_Data

## Data Availability

The data that support the findings of this study are available from the corresponding author upon reasonable request.
