# Supplementary material for: Reversible temporal artery halo sign in polymerase chain reaction–confirmed herpes zoster ophthalmicus without corticosteroid exposure
Source: Rheumatol Adv Pract. 2026 Aug 17;10(3):rkag104. doi: 10.1093/rap/rkag104 (PMC13525336; doi:10.1093/rap/rkag104)
Supplement: Supplementary file 1 [file rkag104_supplementary_data.docx]

Supplementary Table S1: Summary of cases of HZO with positive findings on temporal artery ultrasound.

| Case | Patient | Radiographic findings | Clinical course |
| --- | --- | --- | --- |
| Teodoro et al. | Male, 72 years | Bilateral halo sign +ve  Resolution of halo sign 2 weeks post corticosteroids and valaciclovir  Recurrence of halo sign 3 weeks post discontinuation of halo sign | High dose steroid and PO valaciclovir with deterioration and development of encephalitis. After 2 weeks changed to IV acyclovir with improvement of encephalitis. Headache and visual disturbance recurred following steroid wean and required recommencement. |
| Tsai et al. | Male, 80 years | Right temporal artery halo sign +ve | Clinical course and management not detailed. |
| Yang et al. (this case) | Female, 69 years | Bilateral temporal artery halo sign +ve  Partial radiographic improvement post 2 months valaciclovir therapy  Complete resolution of halo sign 9 months post therapy | 2 weeks oral valaciclovir 1000 mg three times daily. |
